# Comparative assessment of injectable platelet-rich fibrin versus concentrated growth factor for periodontal intrabony defects

**DOI:** 10.6026/973206300220824

**Published:** 2026-02-28

**Authors:** Yasir S Khan, Rishiraj Singh Karki, Tanu Sahney, Shahrukh Khan, Varanasi Haripriya, Pritam Bora

**Affiliations:** 1 Department of Periodontology, Sardar Patel Post Graduate Institute of Dental and Medical Sciences, Lucknow, Uttar Pradesh, India; 2 Department of Dentistry, Government Doon Medical College and Hospital, Dehradun, India; 3 Clinical Practioner, Sri Visweswara Dental Care and Implant Centre, Vishakhapatnam, Andhra Pradesh, India

**Keywords:** Periodontitis, intrabony defects, periodontal regeneration, injectable platelet-rich fibrin (i-PRF), concentrated growth factor (CGF), randomized controlled trial

## Abstract

Periodontal intrabony defects present a therapeutic challenge and direct clinical evidence comparing injectable platelet-rich fibrin (i-PRF) and concentrated growth factor (CGF) as regenerative materials remains limited. This randomized controlled split-mouth clinical trial included 20 patients with 40 comparable three-wall intrabony defects treated by open flap debridement followed by either i-PRF or CGF, with clinical and radiographic parameters evaluated at baseline and 6 months. Both groups showed significant improvements in probing pocket depth, clinical attachment level and radiographic defect fill at 6 months (p<0.001). The CGF group demonstrated significantly greater pocket depth reduction, attachment gain and percentage of radiographic defect fill compared with the i-PRF group (p<0.05). Within the limitations of this study, both materials were effective, although CGF exhibited superior regenerative outcomes and may represent a more predictable option for periodontal intrabony defect regeneration.

## Background:

Periodontitis is a chronic inflammatory disease characterized by the progressive destruction of the tooth-supporting apparatus, including the periodontal ligament, cementum and alveolar bone [1]. This destruction often results in the formation of intrabony defects, which are angular bone defects that present a significant therapeutic challenge. Conventional non-surgical and surgical treatments, such as scaling and root planing or open flap debridement, can arrest disease progression but often result in repair through the formation of a long junctional epithelium rather than true regeneration of lost tissues [2]. The ultimate goal of periodontal therapy for intrabony defects is periodontal regeneration, defined as the complete restoration of the lost periodontium, including new cementum, periodontal ligament and alveolar bone [3]. To achieve this, various biomaterials have been developed and utilized, including bone grafts, guided tissue regeneration (GTR) membranes and biologic agents like enamel matrix derivative [4]. In recent years, autologous platelet concentrates (APCs) have gained considerable attention as a cost-effective and biocompatible source of growth factors essential for tissue healing and regeneration. APCs are broadly classified into generations based on their preparation and composition. Platelet-Rich Fibrin (PRF), a second-generation concentrate overcomes many limitations of its predecessor, Platelet-Rich Plasma (PRP), by eliminating the need for anticoagulants and bovine thrombin [5].

PRF is a dense fibrin matrix that slowly releases a multitude of growth factors, such as platelet-derived growth factor (PDGF), transforming growth factor-beta (TGF-β) and vascular endothelial growth factor (VEGF), over several days [6]. Further modifications of the centrifugation protocol have led to the development of advanced fibrin matrices. Injectable PRF (i-PRF) is a liquid formulation produced by a short, low-speed centrifugation, which maintains a high concentration of platelets, leukocytes and circulating stem cells in a fluid state, allowing for easy injection into defect sites [7]. Conversely, Concentrated Growth Factor (CGF), developed by Sacco, is produced using a centrifuge with a pre-programmed, alternating speed protocol. This unique process is proposed to create a larger, denser and richer fibrin matrix with a higher concentration of growth factors and CD34+ stem cells compared to other PRF-based preparations [8, 9]. Both i-PRF and CGF have shown promising results individually in various regenerative applications, including the treatment of periodontal defects [10, 11]. The fluid nature of i-PRF makes it an excellent carrier for bone graft particles and allows it to adapt intimately to complex defect morphologies, while the robust fibrin scaffold of CGF provides superior space maintenance and a sustained release of biologics. Therefore, it is of interest to compare and report the clinical and radiographic regenerative outcomes of injectable platelet-rich fibrin and concentrated growth factor when used as standalone autologous biomaterials in the surgical management of periodontal intrabony defects.

## Materials and Methods:

This randomized controlled split-mouth clinical trial was conducted in Department of Periodontology, Sardar Patel Post Graduate Institute of Dental and Medical Sciences, Lucknow and included 20 patients, each contributing two comparable, deep (≥4 mm) three-wall intrabony defects. Sample size was calculated based on a previous study comparing different regenerative materials. To detect a mean difference of 1.0 mm in CAL gain between groups with a standard deviation of 0.8 mm.

## Inclusion and exclusion criteria:

Patients aged 25-55 years diagnosed with chronic periodontitis were screened. Inclusion criteria were:

[1] Systemically healthy with no contraindications for periodontal surgery.

[2] Good oral hygiene with a full-mouth plaque score <20%.

[3] Presence of at least two comparable, deep (≥4 mm) three-wall intrabony defects in contralateral quadrants with a probing pocket depth (PPD) ≥6 mm.

[4] Vital teeth with no endodontic involvement.

## Exclusion criteria were:

[1] Smokers or tobacco users.

[2] Pregnant or lactating females.

[3] Systemic diseases known to affect periodontal healing (*e.g.*, uncontrolled diabetes mellitus).

[4] Use of medications known to interfere with periodontal tissues (*e.g.*, corticosteroids, NSAIDs) within the past 3 months.

[5] History of any hematological disorders.

## Pre-surgical phase:

All enrolled patients underwent phase I periodontal therapy, including oral hygiene instructions, full-mouth scaling and root planing. Re-evaluation was performed 8 weeks later to confirm the persistence of the defects and the patient's compliance with oral hygiene.

## Randomization and allocation:

A simple randomization sequence was generated using a coin toss method. The two selected defect sites in each patient were randomly allocated to either Test Group A (i-PRF) or Test Group B (CGF). The allocation was performed by a clinical assistant not involved in the surgical or assessment procedures. The clinical examiner was blinded to the treatment allocation during the 6-month follow-up assessment.

## Surgical procedure:

All surgeries were performed by a single experienced periodontist. Following local anesthesia, a full-thickness mucoperiosteal flap (Modified Widman Flap) was elevated. The intrabony defects were thoroughly debrided of all granulation tissue and the root surfaces were meticulously scaled and planed.

[1] Preparation of i-PRF and CGF: Immediately before flap closure, 10 mL of venous blood was drawn from the patient's antecubital fossa into two sterile vacutainer tubes without anticoagulant.

[2] For Test Group A (i-PRF), the blood tube was immediately centrifuged at 700 rpm (60 g) for 3 minutes (Duo Quattro Centrifuge, Process for PRF, Nice, France). The upper liquid i-PRF layer was collected using a sterile syringe.

[3] For Test Group B (CGF), the blood tube was centrifuged using a Medifuge CGF system (Silfradent, Forlì, Italy) with its specific pre-set program of alternating and controlled speeds (2700 rpm for 2 min, 2400 rpm for 4 min, 2700 rpm for 4 min, 3000 rpm for 3 min). The solid CGF clot located between the red blood cell layer and acellular plasma was separated and gently compressed.

The assigned biomaterial (liquid i-PRF or solid CGF) was placed into the defect, filling it completely. The flaps were then repositioned and secured with 4-0 non-resorbable silk sutures using an interrupted technique.

## Post-surgical care:

Patients were prescribed amoxicillin 500 mg three times daily for 5 days and a 0.12% chlorhexidine gluconate rinse twice daily for 2 weeks. Sutures were removed 10 days post-operatively. Patients were recalled for professional prophylaxis at 1, 3 and 6 months.

## Clinical and radiographic assessment:

All measurements were taken at baseline (immediately before surgery) and 6 months post-operatively by a single calibrated and blinded examiner.

[1] Clinical Parameters: PPD, CAL and Gingival Recession (GR) were measured to the nearest millimetre at six sites per tooth using a UNC-15 periodontal probe.

[2] Radiographic Parameters: Standardized intraoral periapical radiographs were taken using a long-cone paralleling technique with a custom acrylic film holder. Digital images were analyzed using Image J software (NIH, USA). The Radiographic Defect Depth (RDD) was measured from the alveolar crest to the base of the defect. Percentage of Radiographic Defect Fill (RDF %) was calculated as: [(Baseline RDD - 6-month RDD) / Baseline RDD] x 100.

## Statistical analysis:

Data were analyzed using SPSS software version 25.0 (IBM Corp., Armonk, NY). Descriptive statistics (mean ± standard deviation) were calculated for all parameters. The normality of data was confirmed using the Shapiro-Wilk test. A paired t-test was used for intra-group comparisons (baseline vs. 6 months). An independent t-test was used for inter-group comparisons of the changes in parameters. The level of statistical significance was set at p < 0.05.

## Results:

A total of 20 patients (11 females, 9 males; mean age 41.2 ± 6.8 years) completed the 6-month follow-up period. No complications, such as infection, allergy, or flap dehiscence, were observed at any of the 40 treated sites. At baseline, there were no statistically significant differences between the i-PRF and CGF groups for any of the recorded clinical or radiographic parameters, indicating that the groups were well-matched (Table 1). Both treatment modalities resulted in highly significant improvements in all clinical and radiographic parameters at 6 months compared to baseline (p<0.001 for all). In the i-PRF group, mean PPD reduced from 7.40 mm to 3.75 mm and mean CAL improved from 8.35 mm to 5.05 mm. In the CGF group, mean PPD reduced from 7.55 mm to 3.30 mm and mean CAL improved from 8.50 mm to 4.55 mm (Table 2). When comparing the magnitude of improvement between the two groups at 6 months, the CGF group demonstrated statistically significantly greater gains. The mean PPD reduction was 4.25 ± 0.72 mm in the CGF group versus 3.65 ± 0.67 mm in the i-PRF group (p=0.018). Similarly, the mean CAL gain was 3.95 ± 0.83 mm for CGF compared to 3.30 ± 0.73 mm for i-PRF (p=0.025). Radiographically, the percentage of defect fill was significantly higher in the CGF group (65.25 ± 10.31%) than in the i-PRF group (55.80 ± 9.85%) (p=0.011). There was no significant difference in the increase in gingival recession between the two groups (p=0.745) (Table 3).

## Discussion:

The primary objective of this randomized controlled clinical trial was to evaluate and compare the regenerative potential of i-PRF and CGF in the treatment of periodontal intrabony defects. The results of our study clearly demonstrate that both autologous preparations are effective, leading to statistically significant improvements in both clinical and radiographic parameters at 6 months post-surgery. However, when compared directly, CGF yielded superior outcomes in terms of PPD reduction, CAL gain and radiographic bone fill. The significant improvements observed within both groups are consistent with the established biological rationale for using platelet concentrates in tissue regeneration. The fibrin matrix in both i-PRF and CGF acts as a bioactive scaffold, promoting cell migration, proliferation and differentiation, while the entrapped platelets and leukocytes serve as a sustained-release reservoir for a cocktail of growth factors crucial for wound healing and angiogenesis [6, 12]. The clinical success of i-PRF in our study aligns with findings from previous investigations that reported significant PPD reduction and CAL gain when i-PRF was used alone or in combination with bone grafts for treating intrabony defects [10, 13]. The novel finding of this study is the statistically significant superiority of CGF over i-PRF. The mean CAL gain in the CGF group was 3.95 mm, which is not only greater than the 3.30 mm achieved with i-PRF but is also clinically relevant, representing a substantial restoration of lost periodontal support. This enhanced performance of CGF may be attributed to its unique preparation protocol. The proprietary alternating centrifugation speed is thought to more effectively separate blood components, resulting in a larger and denser fibrin matrix that is richer in growth factors, platelets and leukocytes, including CD34+ stem cells, compared to PRF prepared with a fixed-speed centrifuge [8, 14]. A denser fibrin scaffold could provide better space maintenance within the defect, preventing soft tissue collapse and allowing more time and space for bone-forming cells to populate the area. Furthermore, a higher concentration of growth factors may create a more potent chemotactic and mitogenic stimulus, accelerating the regenerative cascade [9, 15].

Radiographic analysis further corroborated the clinical findings. The CGF group achieved a mean defect fill of 65.25%, which was significantly higher than the 55.80% in the i-PRF group. This indicates a more robust and predictable bone formation with CGF. While two-dimensional radiography has inherent limitations, the use of a standardized technique and digital analysis provides a reliable quantitative measure of bone level changes. It is important to interpret the results within the context of the study's limitations. Firstly, the 6-month follow-up period, while common in periodontal studies, may not be sufficient to fully assess the long-term stability of the regenerated tissues. Secondly, this study lacked a true negative control group (*e.g.*, open flap debridement alone), although the split-mouth design allows each patient to serve as their own control, minimizing inter-patient variability. Thirdly, the assessment of regeneration was based on clinical and radiographic parameters, not histology, which remains the gold standard for confirming the nature of newly formed tissue. Future studies with longer follow-up periods, three-dimensional cone-beam computed tomography (CBCT) analysis and, where possible, histological evaluation are warranted to confirm these findings. Despite these limitations, this study provides valuable clinical evidence supporting the use of CGF in periodontal regenerative surgery. The preparation is simple, cost-effective and uses a fully autologous material, eliminating the risks of disease transmission or immune reactions associated with allografts or xenografts. The observed superiority of CGF suggests that the specific centrifugation protocol plays a critical role in optimizing the regenerative potential of platelet concentrates.

## Conclusion:

Both Injectable Platelet-Rich Fibrin and Concentrated Growth Factor were found to be safe and effective treatment modalities for periodontal intrabony defects, leading to significant improvements in clinical and radiographic outcomes. However, Concentrated Growth Factor demonstrated a marginal but statistically significant advantage over Injectable Platelet-Rich Fibrin in promoting probing pocket depth reduction, clinical attachment level gain and radiographic defect fill. Thus, we show that CGF may be a more predictable and potent autologous biomaterial for achieving periodontal regeneration.

## Figures and Tables

**Table 1:** Baseline clinical and radiographic parameters (Mean ± SD)

**Parameter**	**Test Group A (i-PRF) (n=20)**	**Test Group B (CGF) (n=20)**	p-value*
PPD (mm)	7.40±0.94	7.55±0.89	0.612
CAL (mm)	8.35±1.14	8.50±1.05	0.678
GR (mm)	0.95±0.60	0.95±0.51	1
RDD (mm)	5.20±0.85	5.35±0.93	0.601

**Table 2:** Intra-group comparison of clinical and radiographic parameters at baseline and 6 months (Mean ± SD)

**Parameter**	**Group**	**Baseline**	**6 Months**	**Change**	p-value**
PPD (mm)	i-PRF	7.40±0.94	3.75±0.64	3.65±0.67	<0.001
	CGF	7.55±0.89	3.30±0.66	4.25±0.72	<0.001
CAL (mm)	i-PRF	8.35±1.14	5.05±0.94	3.30±0.73	<0.001
	CGF	8.50±1.05	4.55±0.94	3.95±0.83	<0.001
RDD (mm)	i-PRF	5.20±0.85	2.30±0.57	2.90±0.55	<0.001
	CGF	5.35±0.93	1.86±0.58	3.49±0.62	<0.001

**Table 3:** Inter-group comparison of clinical and radiographic changes at 6 months (Mean ± SD)

**Parameter Change**	**Test Group A (i-PRF) (n=20)**	**Test Group B (CGF) (n=20)**	p-value*
PPD Reduction (mm)	3.65 ± 0.67	4.25 ± 0.72	0.018
CAL Gain (mm)	3.30 ± 0.73	3.95 ± 0.83	0.025
GR Increase (mm)	0.35 ± 0.49	0.30 ± 0.47	0.745
RDD Reduction (mm)	2.90 ± 0.55	3.49 ± 0.62	0.003
RDF (%)	55.80 ± 9.85	65.25 ± 10.31	0.011
